# Supplementary material for: Causal Relationship between Aging and Anorexia Nervosa: A White-Matter-Microstructure-Mediated Mendelian Randomization Analysis
Source: Biomedicines. 2024 Aug 16;12(8):1874. doi: 10.3390/biomedicines12081874 (PMC11351342; doi:10.3390/biomedicines12081874)
Supplement: Supplementary file 1 [file biomedicines-12-01874-s001.zip › Supplementary Figure/Figure S4/Singe SNP plot of MR analysis for TL to ubm-b-1895.pdf]

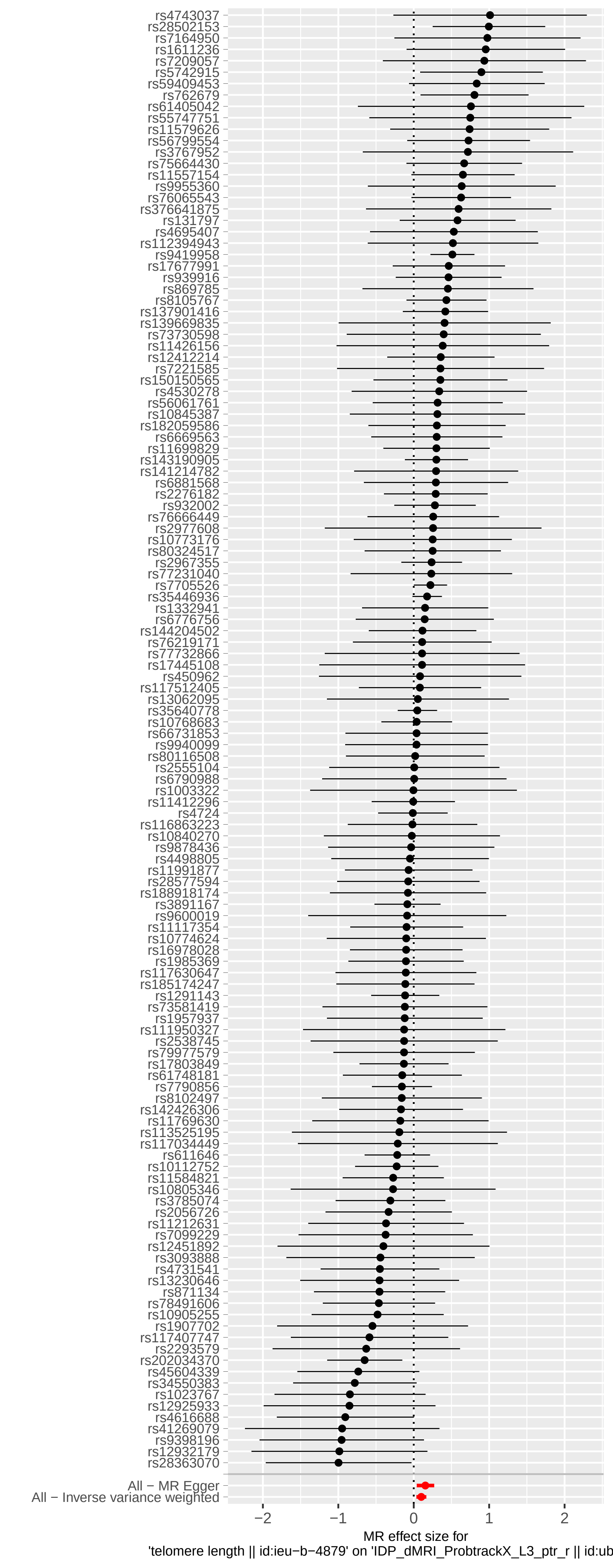
rs4743037
rs28502153
rs7164950
rs1611236
rs7209057
rs5742915
rs59409453
rs762679
rs61405042
rs55747751
rs11579626
rs56799554
rs3767952
rs75664430
rs11557154
rs9955360
rs76065543
rs376641875
rs131797
rs4695407
rs112394943
rs9419958
rs17677991
rs939916
rs869785
rs8105767
rs137901416
rs139669835
rs73730598
rs11426156
rs12412214
rs7221585
rs150150565
rs4530278
rs56061761
rs10845387
rs182059586
rs6669563
rs11699829
rs143190905
rs141214782
rs6881568
rs2276182
rs932002
rs76666449
rs2977608
rs10773176
rs80324517
rs2967355
rs77231040
rs7705526
rs35446936
rs1332941
rs6776756
rs144204502
rs76219171
rs77732866
rs17445108
rs450962
rs117512405
rs13062095
rs35640778
rs10768683
rs66731853
rs9940099
rs80116508
rs2555104
rs6790988
rs1003322
rs11412296
rs4724
rs116863223
rs10840270
rs9878436
rs4498805
rs11991877
rs28577594
rs188918174
rs3891167
rs9600019
rs11117354
rs10774624
rs16978028
rs1985369
rs117630647
rs185174247
rs1291143
rs73581419
rs1957937
rs111950327
rs2538745
rs79977579
rs17803849
rs61748181
rs7790856
rs8102497
rs142426306
rs11769630
rs113525195
rs117034449
rs611646
rs10112752
rs11584821
rs10805346
rs3785074
rs2056726
rs11212631
rs7099229
rs12451892
rs3093888
rs4731541
rs13230646
rs871134
rs78491606
rs10905255
rs1907702
rs117407747
rs2293579
rs202034370
rs45604339
rs34550383
rs1023767
rs12925933
rs4616688
rs41269079
rs9398196
rs12932179
rs28363070
All − MR Egger
All − Inverse variance weighted
−2
−1
0
1
2
MR effect size for
'telomere length || id:ieu−b−4879' on 'IDP_dMRI_ProbtrackX_L3_ptr_r || id:ub
